# Associations of Fully Automated CSF and Novel Plasma Biomarkers With Alzheimer Disease Neuropathology at Autopsy

**DOI:** 10.1212/WNL.0000000000012513

**Published:** 2021-09-21

**Authors:** Michel J. Grothe, Alexis Moscoso, Nicholas J. Ashton, Thomas K. Karikari, Juan Lantero-Rodriguez, Anniina Snellman, Henrik Zetterberg, Kaj Blennow, Michael Schöll, for the Alzheimer's Disease Neuroimaging Initiative

**Affiliations:** From the Unidad de Trastornos del Movimiento (M.J.G.), Servicio de Neurología y Neurofisiología Clínica, Instituto de Biomedicina de Sevilla, Hospital Universitario Virgen del Rocío/CSIC/Universidad de Sevilla, Spain; Department of Psychiatry and Neurochemistry (M.J.G., A.M., N.J.A., T.K.K., J.L.-R., A.S., H.Z., K.B., M.S.), Institute of Neuroscience and Physiology, The Sahlgrenska Academy, and Wallenberg Centre for Molecular and Translational Medicine (M.J.G., A.M., N.J.A., M.S.), University of Gothenburg, Sweden; King's College London (N.J.A.), Institute of Psychiatry, Psychology and Neuroscience, Maurice Wohl Clinical Neuroscience Institute; NIHR Biomedical Research Centre for Mental Health and Biomedical Research Unit for Dementia at South London and Maudsley NHS Foundation (N.J.A.), London, UK; Turku PET Centre (A.S.), University of Turku, Finland; Clinical Neurochemistry Laboratory (H.Z., K.B.), Sahlgrenska University Hospital, Mölndal, Sweden; Department of Neurodegenerative Disease (H.Z., M.S.), UCL Institute of Neurology; and UK Dementia Research Institute at UCL (H.Z.), London, UK.

## Abstract

**Objective:**

To study CSF biomarkers of Alzheimer disease (AD) analyzed by fully automated Elecsys immunoassays compared to neuropathologic gold standards and to compare their accuracy to plasma phosphorylated tau (p-tau181) measured with a novel single molecule array method.

**Methods:**

We studied antemortem Elecsys-derived CSF biomarkers in 45 individuals who underwent standardized postmortem assessments of AD and non-AD neuropathologic changes at autopsy. In a subset of 26 participants, we also analyzed antemortem levels of plasma p-tau181 and neurofilament light (NfL). Reference biomarker values were obtained from 146 amyloid-PET–negative healthy controls (HC).

**Results:**

All CSF biomarkers clearly distinguished pathology-confirmed AD dementia (n = 27) from HC (area under the curve [AUC] 0.86–1.00). CSF total tau (t-tau), p-tau181, and their ratios with β-amyloid_1-42_ (Aβ_1-42_) also accurately distinguished pathology-confirmed AD from non-AD dementia (n = 8; AUC 0.94–0.97). In pathology-specific analyses, intermediate to high Thal amyloid phases were best detected by CSF Aβ_1-42_ (AUC [95% confidence interval] 0.91 [0.81–1]), while intermediate to high scores for Consortium to Establish a Registry for Alzheimer's Disease neuritic plaques and Braak tau stages were best detected by CSF p-tau181 (AUC 0.89 [0.79–0.99] and 0.88 [0.77–0.99], respectively). Optimal Elecsys biomarker cutoffs were derived at 1,097, 229, and 19 pg/mL for Aβ_1-42_, t-tau, and p-tau181. In the plasma subsample, both plasma p-tau181 (AUC 0.91 [0.86–0.96]) and NfL (AUC 0.93 [0.87–0.99]) accurately distinguished those with pathology-confirmed AD (n = 14) from HC. However, only p-tau181 distinguished AD from non-AD dementia cases (n = 4; AUC 0.96 [0.88–1.00]) and showed a similar, although weaker, pathologic specificity for neuritic plaques (AUC 0.75 [0.52–0.98]) and Braak stage (AUC 0.71 [0.44–0.98]) as CSF p-tau181.

**Conclusion:**

Elecsys-derived CSF biomarkers detect AD neuropathologic changes with very high discriminative accuracy in vivo. Preliminary findings support the use of plasma p-tau181 as an easily accessible and scalable biomarker of AD pathology.

**Classification of Evidence:**

This study provides Class II evidence that fully automated CSF t-tau and p-tau181 measurements discriminate between autopsy-confirmed AD and other dementias.

The recent guidelines of the National Institute on Aging–Alzheimer’s Association (NIA-AA) Research Framework now define Alzheimer disease (AD) as a biological entity for which an in vivo diagnosis is no longer based solely on clinical diagnostic criteria but requires supporting evidence from PET or fluid biomarkers of AD-typical β-amyloid (Aβ) and tau pathology.^1-3^ In contrast to PET, bodily fluid–based measurements can provide different molecular biomarkers from a single assessment and are more cost effective, widely attainable, and not limited by radiation exposure. Yet, the international Alzheimer’s Association Quality Control Program for CSF and blood biomarkers has shown large variability (>15%) of the commonly used manual plate-based ELISAs for AD biomarker quantification in CSF across several laboratories.^4^ A major step toward widespread clinical use of CSF biomarkers has been the development of standardized measurements through fully automated platforms with high test-retest reliability (<5%) and low laboratory- and kit-associated variability such as the Roche Elecsys electrochemiluminescence immunoassays,^5^ which show excellent concordance with the manual ELISAs^6^ and have been well validated against Aβ PET.^7-10^

The recent development of assays to measure phosphorylated tau in blood offers an alternative opportunity to assess AD pathology in a cost-effective, highly accessible, and scalable manner. Plasma concentrations of tau phosphorylated at threonine 181 (p-tau181) correlate highly with CSF measures of p-tau181 and with PET measures of Aβ and tau pathology^11-15^ and have been shown to distinguish between AD and other neurodegenerative disorders with high diagnostic accuracy comparable to that of CSF and PET-based measures of tau pathology.^11-13,16^

However, relatively few studies have thus far aimed to validate the established CSF^17-21^ or the novel plasma p-tau181 biomarkers^11,22^ against neuropathologic gold standards. Specifically, to date, no neuropathologic validation exists of the fully automated Elecsys-derived Aβ and tau biomarker measurements, and currently recommended cutoffs for these standardized measures are based on concordance studies with Aβ-PET or clinical criteria.^7-10,23^

In this study, we examined antemortem Elecsys-derived CSF biomarkers in relation to AD neuropathology assessed at autopsy in the same individuals. In preliminary analyses on a smaller subset of participants, we also analyzed antemortem levels of plasma p-tau181 and neurofilament light (NfL). Specifically, we first studied the diagnostic accuracy of the fluid biomarkers for distinguishing pathology-confirmed AD dementia cases from Aβ-PET-negative healthy controls and dementia cases without AD pathology at autopsy. We then assessed the specific associations of the different Aβ and tau biomarkers with distinct aspects of AD neuropathology, including established neuropathologic rating scales for regional extension of Aβ pathology (Thal phases), cortical density of diffuse and neuritic Aβ plaques (Consortium to Establish a Registry for Alzheimer's Disease [CERAD] score), and regional extension of neurofibrillary tangle tau pathology (Braak stages). We derived pathology-specific biomarker cutoffs that best separated individuals with absent to low levels from those with intermediate to high levels of the respective AD neuropathologic correlate. Finally, we assessed the sensitivity of the biomarkers for the presence of common non-AD pathologies at autopsy, including cerebral amyloid angiopathy (CAA), Lewy body (LB) pathology, and limbic TAR DNA-binding protein 43 (TDP-43) pathology.

## Methods

### Data Source

Data used in the preparation of this article were obtained from the Alzheimer's Disease Neuroimaging Initiative (ADNI) database. The ADNI is a public-private partnership that was launched in 2003 with the primary goal of testing whether neuroimaging and other biological markers can be used to track disease progression in AD. The ADNI website provides up-to-date information.

### Standard Protocol Approvals, Registrations, and Patient Consents

Data collection and sharing were approved by the Institutional Review Board of each institution participating in ADNI. All participants provided written informed consent in accordance with the Declaration of Helsinki and its later amendments.

### Study Participants

In the present study, we used data from the subsample of ADNI participants who had been followed up to autopsy for standardized neuropathologic examinations (neuropathology data freeze April 12, 2018). From this ADNI autopsy cohort, we identified 45 participants who had available antemortem CSF measurements, with an average time difference between lumbar puncture and death of 2.9 (SD 1.9, interquartile range [IQR] 1.7–3.7, minimum–maximum 0.4–8.7) years (see supplementary Figure S1 for a flowchart of patient selection; data available from Dryad: doi.org/10.5061/dryad.n2z34tmwr). Participants were recruited between 2005 and 2013 and were followed up to autopsy between 2008 and 2017. Thirty-five participants were diagnosed with AD dementia, 6 with mild cognitive impairment, and 4 as cognitively normal at their last clinical evaluation (on average 1.8 [SD 1.6, IQR 0.7–2.7] years before death) according to standard diagnostic criteria used in the ADNI study. A subsample of 26 participants (18 with AD, 4 with mild cognitive impairment, 4 cognitively normal) also had available plasma measurements that were used for a head-to-head comparison of CSF and plasma biomarkers. Average time difference between CSF and plasma sampling was 1.2 (SD 1.2, IQR 0.8–1.2) years in this subsample.

To derive reference values for the biomarker measurements, we also included data from a control group of 146 cognitively normal individuals with a negative Aβ-PET scan who had available CSF and plasma measurements. This Aβ-PET–negative control group was selected on the basis of a global cortical [^18^F]florbetapir-PET signal <12 Centiloids,^24^ which was calculated from standardized uptake value ratios with equations derived by the ADNI PET Core.^15^

### Neuropathologic Examination

All neuropathologic assessments were performed by the same neuropathologist (Dr. Nigel Cairns) at the central laboratory of the ADNI Neuropathology Core at the Knight Alzheimer's Disease Research Center, Washington University School of Medicine, St. Louis, MO (directed by Dr. John C. Morris), which provides uniform neuropathologic assessments of deceased ADNI participants.^25^ Neuropathologic evaluations assessed a wide range of AD neuropathologic lesions and common non-AD pathologies following NIA-AA guidelines for the neuropathologic assessment of AD,^26^ which are itemized in the Neuropathology Data Form Version 10 of the National Alzheimer Coordinating Center.

The principal neuropathologic outcome measures of the present study were focused on established rating scales for different aspects of AD neuropathologic change (ADNC), including Thal phases of regional distribution of Aβ plaques (A), Braak stages of tau neurofibrillary tangle pathology (B), and CERAD scores for density of neuritic (C) and diffuse (D) plaques.^26^ Following the NIA-AA guidelines, Thal phases (0–5) and Braak stages (0–6) were converted to A and B scores, so that all neuropathologic rating scales (A–D) are scored on a common semiquantitative 4-point scale from absent (0) to low (1), intermediate (2), and high (3). The A-B-C scores were further collapsed into a 4-point scale ADNC composite score according to NIA-AA neuropathologic criteria, in which scores ≥2 correspond to a pathologic diagnosis of AD.^26,27^ Thus, ADNC composite scores were used to classify patients with a clinical diagnosis of AD dementia (n = 35) into pathology-confirmed AD dementia (ADNC score ≥2; n = 27) and non-AD dementia (ADNC score ≤1; n = 8) groups. Primary neuropathologic diagnoses in the non-AD dementia group included LB disease (n = 4), hippocampal sclerosis (n = 2), argyrophylic grain disease (n = 1), and frontotemporal lobar degeneration with TDP-43 inclusions (n = 1). Although CERAD scores for density of diffuse plaques (D) are not used for calculating the ADNC composite score, they were included in pathology-specific analyses to allow assessment of biomarker-specific associations with neuritic vs diffuse Aβ plaques. Neuritic plaques are considered pathologically advanced forms of Aβ plaques and can be distinguished from diffuse plaques by the presence of dystrophic neurites, which typically also exhibit immunoreactivity for phosphorylated tau.^26,28^

In addition to AD-specific neuropathology, we assessed common comorbid non-AD pathologies, including CAA, LB, and TDP-43 pathology. Presence of CAA was assessed in parenchymal and leptomeningeal vessels and scored on a semiquantitative 4-point scale based on global brain area involvement (absent to widespread). Evidence of LB pathology was assessed according to modified McKeith criteria,^26,29^ and assessment of TDP-43 pathology followed a regional evaluation of TDP-43–immunoreactive inclusions in the spinal cord, amygdala, hippocampus, entorhinal cortex/inferior temporal gyrus, and frontal neocortex.^30^ For the purpose of the present study, all neuropathologic assessment scales/scores of non-AD pathologies were dichotomized into 0 = absent and 1 = present categories.

More detailed information on the implementation and operational definitions of the different neuropathologic rating scales is provided in the coding guidebook of the National Alzheimer Coordinating Center Neuropathology Data Form.

### CSF Biomarkers

Available antemortem CSF samples were analyzed for peptide levels of Aβ_1-42_, total tau (t-tau), and p-tau181 with the fully automated Roche Elecsys electrochemiluminescence immunoassays on a cobas e601 instrument (Roche Diagnostics, Indianapolis, IN) according to the kit manufacturer's instructions. The lower and upper technical limits for the biomarkers are 200 to 1,700 pg/mL for Aβ_1-42_, 80 to 1,300 pg/mL for t-tau, and 8 to 120 pg/mL for p-tau181.

In the present study, we also included Aβ_1-42_ values beyond the upper technical limit, which are provided on the basis of an extrapolation of the calibration curve. However, note that the use of these values is restricted to exploratory research purposes, and they should not be used for clinical decision-making.

### Plasma Biomarkers

Blood samples were collected and processed according to the ADNI protocol^31^ and analyzed at the Clinical Neurochemistry Laboratory, University of Gothenburg, Mölndal, Sweden. Plasma p-tau181 concentration was measured with a novel assay developed in-house on a single molecule array HD-X (Quanterix, Billerica, MA) instrument, as described previously.^12,14^

For comparison, we also included plasma NfL as a biomarker for general neurodegeneration, which is not specific for AD pathology.^32^ Plasma NfL also was measured using the single molecule array platform as previously described.^33,34^

### Statistical Analysis

In a first analysis, we used Mann-Whitney *U* tests to study the difference in biomarker levels between patients with pathology-confirmed AD dementia (ADNC ≥2), Aβ-PET–negative control participants, and patients with a clinical diagnosis of AD dementia but without neuropathologic evidence of AD pathology (ADNC score ≤1, non-AD dementia). The accuracy with which the biomarkers could discriminate between these groups was tested using the area under the curve (AUC) in receiver operating characteristic (ROC) curve analysis, and optimal biomarker cutoffs for group separation were derived from the value that maximizes the Youden index (sensitivity + specificity − 1).

In a second set of analyses, we studied pathology-specific associations of the different biomarkers with distinct aspects of ADNC. Given that biomarkers are supposed to reflect specific pathologic processes regardless of their potential clinical consequences,^3^ these association analyses were carried out across pooled diagnostic groups to increase pathologic variance in the sample. Associations between fluid biomarkers and neuropathologic measures were examined with 2 complementary analyses. First, Spearman partial correlations, adjusted for the time interval between biomarker collection and death, were calculated for the association between each fluid biomarker and the different semiquantitative neuropathologic rating scales. Second, the ability of the biomarkers to detect intermediate to high degrees of the different ADNCs, as well as the presence of non-AD pathologies, was quantified with ROC curve analysis as described above. Similar to the ADNC composite score, the 4-point semiquantitative rating scales (A–D) were dichotomized into high and low categories for this analysis according to a distinction of intermediate/high (2/3) vs absent/low (0/1) degrees of pathology.

All analyses were conducted separately for the full sample with available CSF data (n = 45) and the subsample of 26 participants who additionally had plasma measurements. Statistical significance threshold was set at *p* < 0.05.

### Data Availability

Data used in this study have been made publicly available by the ADNI in the Laboratory of Neuro Imaging database.

## Results

### Sample Characteristics

Demographic, clinical, and neuropathologic characteristics of the analyzed sample are summarized in Table 1. Average time difference between biofluid collection and time of death was 2.9 (SD 1.9, IQR 1.7–3.7, minimum–maximum 0.4–8.7) years. At the last assessment before death, a clinical diagnosis of AD dementia was given in the majority of individuals (78%). Intermediate to high neuropathologic change scores were considerably more frequent than absent to low scores for all neuropathologic rating scales, especially for Thal phases (A) and diffuse plaque (D) scores, and 71% of all cases had intermediate to high ADNC composite scores, qualifying for a neuropathologic diagnosis of AD.

**Table 1:**
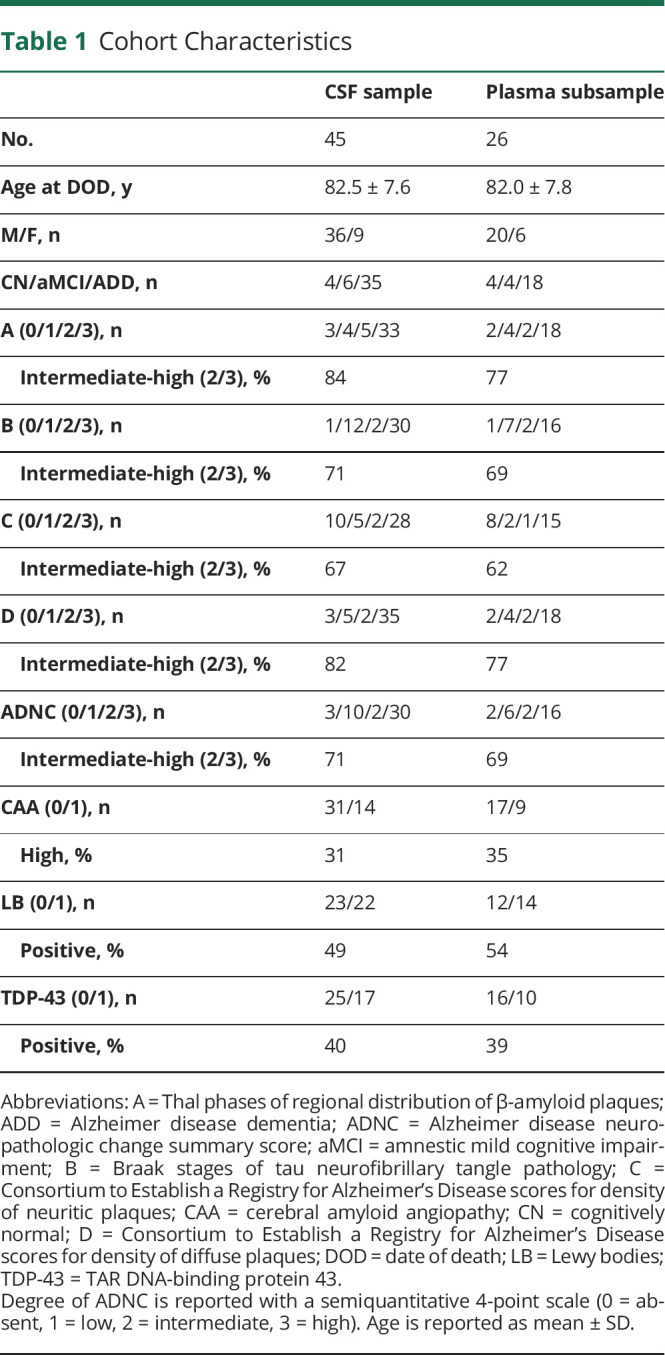
Cohort Characteristics

Overall, the different neuropathologic rating scales were highly interrelated, specifically A and D scores (Spearman ρ = 0.92) as well as B and C scores (ρ = 0.94), whereas the associations between these neuropathologic categories were weaker (A-B: ρ = 0.86; A-C: ρ = 0.84; D-B: ρ = 0.77; D-C: ρ = 0.76; all *p* < 0.001).

With regard to non-AD pathologies, 31% of all cases exhibited intermediate to high levels of CAA, which were associated with AD neuropathology scores, most notably A scores (CAA-A: ρ = 0.40, *p* = 0.007; CAA-B: ρ = 0.37, *p* = 0.013; CAA-C: ρ = 0.27, *p* = 0.074; CAA-D: ρ = 0.36; *p* = 0.016). About half of the sample (49%) had evidence of LB pathology, and 40% had evidence of TDP-43 pathology, but neither was associated with any AD neuropathology score (all ρ < 0.22, *p* > 0.14).

Demographic, clinical, and neuropathologic characteristics of the subsample with available plasma measurements were comparable to those of the full sample (Table 1).

### Discriminative Accuracy of Fluid Biomarkers for Distinguishing Those With Pathology-Confirmed AD Dementia From Healthy Controls and Those With Non-AD Dementia

**Figure 1:**
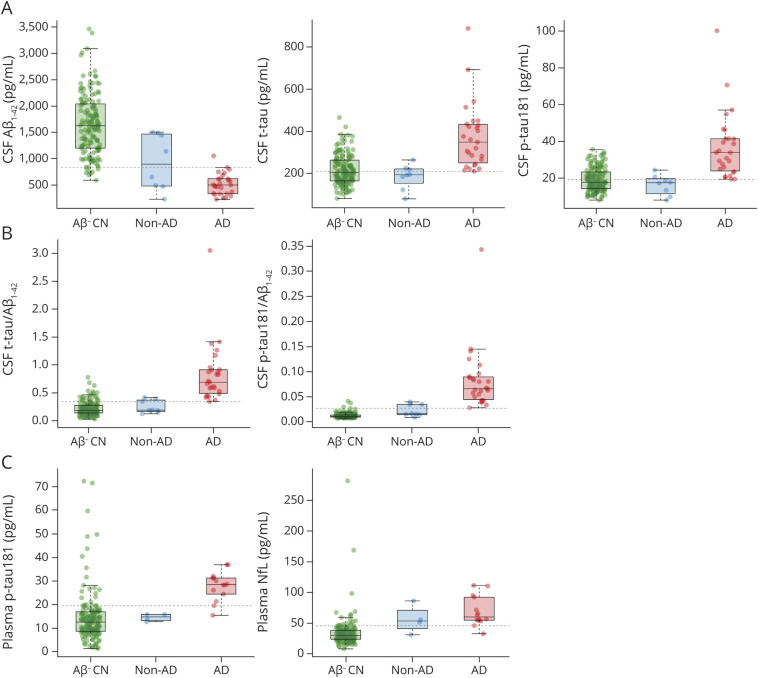
Fluid Biomarker Levels in Pathology-Confirmed AD Dementia, Non-AD Dementia, and Aβ-PET–Negative Healthy Controls (A) CSF levels of β-amyloid_1-42_ (Aβ_1-42_), total tau (t-tau), and tau phosphorylated at threonine 181 (p-tau181). (B) CSF-based ratios of t-tau to Aβ_1-42_ and p-tau181 to Aβ_1-42_. (C) Plasma levels of p-tau181 and neurofilament light (NfL). Dashed lines represent biomarker cutoffs corresponding to the optimal cutoffs determined in the receiver operating characteristic analysis of those with pathology-confirmed Alzheimer disease (AD) dementia vs amyloid-negative cognitively normal (CN). Aβ- CN = CN individuals with a negative Aβ-PET scan; AD = patients with pathology-confirmed AD dementia (AD neuropathologic change summary score ≥2); non-AD = patients with a clinical diagnosis of AD dementia but without neuropathologic evidence of AD pathology (AD neuropathologic change summary score ≤1).

**Figure 2:**
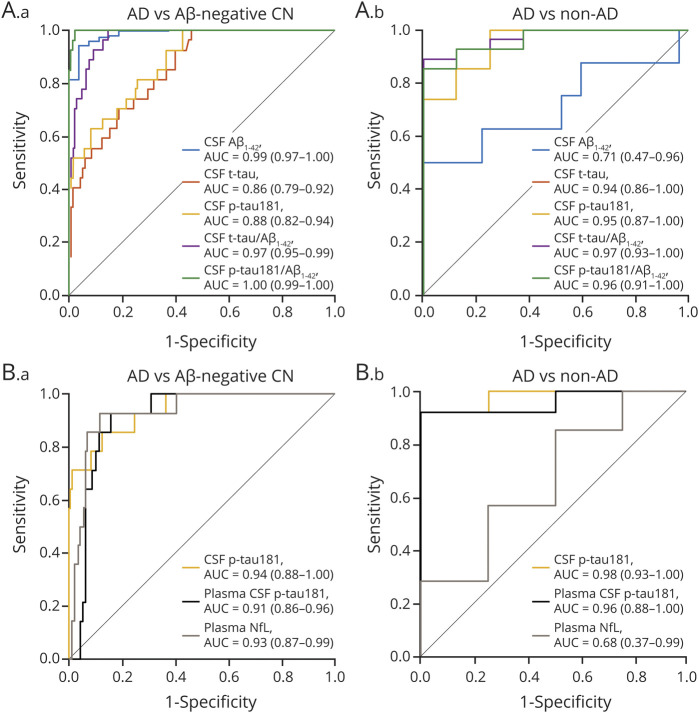
ROC Curves for Distinguishing Pathology-Confirmed AD Dementia From Non-AD Dementia and Aβ-PET–Negative Healthy Controls Receiver operating characteristic (ROC) curves showing the performance of Elecsys CSF biomarkers (A) and plasma biomarkers compared to CSF tau phosphorylated at threonine 181 (p-tau181) (B) for the discrimination of pathology-confirmed Alzheimer disease (AD) dementia from β-amyloid (Aβ)-PET–negative healthy controls (A.a and B. a) and non-AD dementia (A.b and B.b). Areas under the curve (AUC) and 95% confidence interval are reported in the inset of each panel. CN = cognitively normal; NfL = neurofilament light; t-tau = total tau.

In the full sample, all Elecsys CSF biomarkers were significantly different between patients with pathology-confirmed AD dementia (n = 27) and Aβ-PET–negative healthy controls (n = 146; all *p* < 0.001; Figure 1) and differentiated between these groups with very high AUC values ranging from 0.86 (t-tau) to 1.00 (p-tau181/Aβ_1-42_ ratio) (Figure 2). Optimal biomarker cutoffs for this differentiation were 838 pg/mL for Aβ_1-42,_ 211 pg/mL for t-tau, 19.3 pg/mL for p-tau181, 0.34 for t-tau/Aβ_1-42_, and 0.027 for p-tau181/Aβ_1-42_. Elecsys t-tau and p-tau181 levels, as well as their ratios with Aβ_1-42_, were also markedly higher in pathology-confirmed AD compared to non-AD dementia (n = 8; all *p* < 0.001; Figure 1) and separated these groups with very high accuracy (AUC 0.94–0.97); differences in Aβ_1-42_ levels, however, were only marginally significant (*p* = 0.07, AUC [95% confidence interval] 0.71 [0.47–0.96]) (Figure 2).

In the subsample with available blood plasma measurements, plasma p-tau181 levels showed a similarly pronounced group difference and high discriminative accuracy for distinguishing those with pathology-confirmed AD (n = 14) from Aβ-PET–negative controls (*p* < 0.001, AUC 0.91 [0.86–0.96], optimal cutoff 19.5 pg/mL) and from non-AD dementia cases (n = 4; *p* = 0.003, AUC = 0.96 [0.88–1.00]; Figure 2). Plasma NfL showed similarly good discrimination of pathology-confirmed AD from Aβ-PET–negative controls (*p* < 0.001, AUC 0.93 [0.87–0.99], optimal cutoff 45.7 pg/mL) but not from non-AD dementia cases (*p* = 0.33, AUC 0.68 [0.37–0.99]).

### Fluid Biomarker Associations With Different AD Neuropathologic Rating Scales and Presence of Non-AD Pathologies

**Figure 3:**
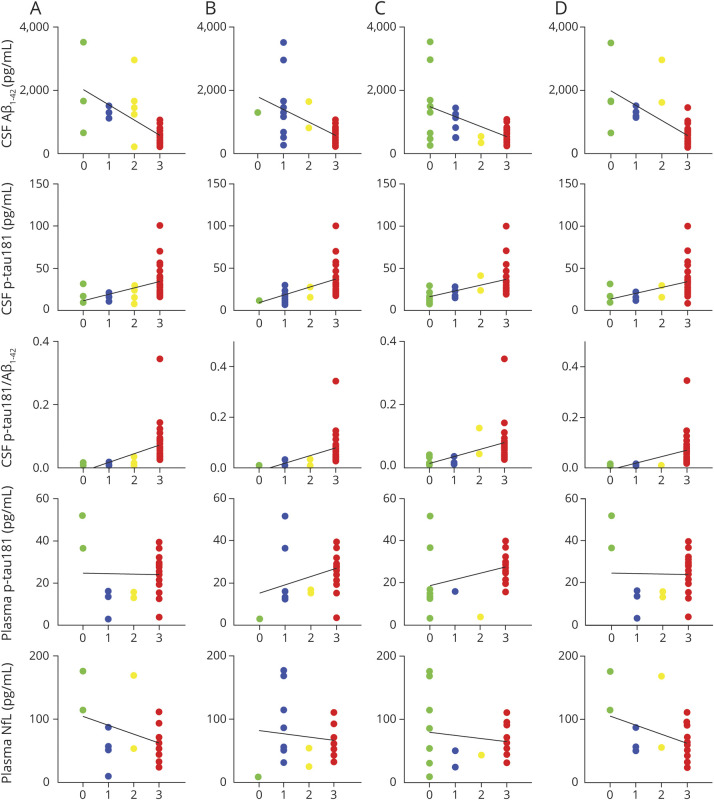
Distribution of Fluid Biomarker Levels Across Distinct ADNC Scores A–D) Four-point semiquantitative scales (0 = absent, 1 = low, 2 = intermediate, and 3 = high) describing Thal phases of regional distribution of β-amyloid plaques (Aβ) (A), Braak stages of tau neurofibrillary tangle pathology (B), Consortium to Establish a Registry for Alzheimer's Disease (CERAD) scores for density of neuritic plaques (C), and CERAD scores for density of diffuse plaques (D). Solid black lines represent linear regression trends. Corresponding Spearman correlation coefficients are listed in Table 2. ADNC = Alzheimer disease neuropathologic change; NfL = neurofilament light; p-tau181 = tau phosphorylated at threonine 181.

The distribution of biomarker values across the different AD neuropathologic rating scales is displayed in Figure 3, and Table 2 lists the corresponding Spearman correlation coefficients. In the full sample, all individual Elecsys CSF biomarkers were significantly associated with the different AD neuropathologic rating scales, but neuropathologic correlations for Aβ_1-42_ were strongest with diffuse plaque scores (D) and Thal phase (A), whereas those for t-tau and p-tau181 were strongest with neuritic plaques (C) and Braak stage (B). However, for all neuropathologic scores, the strongest correlations were observed for the t-tau/Aβ_1-42_ and p-tau181/Aβ_1-42_ ratios.

**Table 2:**
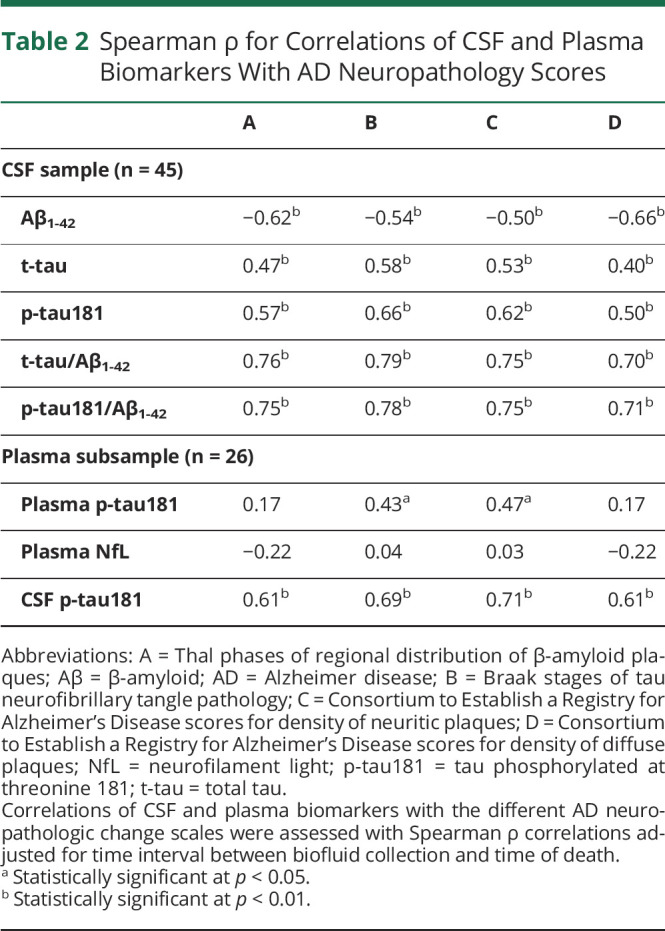
Spearman ρ for Correlations of CSF and Plasma Biomarkers With AD Neuropathology Scores

**Figure 4:**
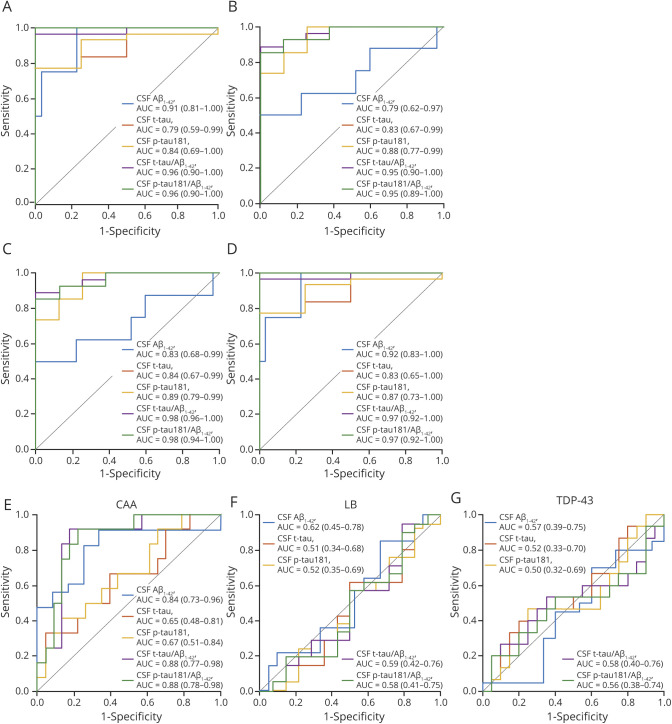
ROC Curves of Elecsys CSF Biomarkers for Detecting ADNCs and Presence of Non-AD Pathologies Receiver operating characteristic (ROC) curves showing the performance of Elecsys CSF biomarkers for detecting intermediate to high degrees of different Alzheimer disease neuropathologic changes (ADNCs). (A) Thal phases of regional distribution of β-amyloid (Aβ) plaques. (B) Braak stages of tau neurofibrillary tangle pathology. (C) Consortium to Establish a Registry for Alzheimer's Disease (CERAD) scores for density of neuritic plaques. (D) CERAD scores for density of diffuse plaques. Presence of (E) cerebral amyloid angiopathy (CAA), (F) Lewy body (LB) pathology, and (G) TAR DNA-binding protein 43 (TDP-43) pathology. AUC = area under the curve; p-tau181 = tau phosphorylated at threonine 181; t-tau = total tau.

Correspondingly, ROC analyses indicated relatively high accuracy for all individual Elecsys CSF biomarkers to differentiate between high and low degrees of the different ADNC scores (Figure 4 and Table 3). High and low degrees of Thal phase (A) and diffuse plaque scores (D) were best differentiated by Aβ_1-42_ levels (AUC 0.91 [0.81–1] and 0.92 [0.83–1], respectively), yielding an optimal cutoff of 1,097 pg/mL for both analyses. High and low degrees of Braak stage (B) and neuritic plaque scores (C) were best differentiated by p-tau181 levels (AUC 0.88 [0.77–0.99] and 0.89 [0.79–0.99], respectively), yielding an optimal cutoff of 19.1 pg/mL for both analyses. The t-tau cutoff that best differentiated between high and low Braak stage (B) was 229 pg/mL, and the same optimal cutoff was found for neuritic plaques (C), although lower cutoffs of 221 and 210 pg/mL yielded identical Youden indices in this ROC analysis. For all neuropathologic rating scales, high and low degrees of pathology were best differentiated by the t-tau/Aβ_1-42_ and p-tau181/Aβ_1-42_ ratios (AUC 0.95–0.98), for which cutoffs of 0.27 for t-tau/Aβ_1-42_ and 0.016 for p-tau181/Aβ_1-42_ yielded the best separation for Thal phases (A) and diffuse plaques (D), whereas higher cutoffs of 0.42 for t-tau/Aβ_1-42_ and 0.041 for p-tau181/Aβ_1-42_ yielded the best separation for Braak stages (B) and neuritic plaques (C).

**Table 3:**
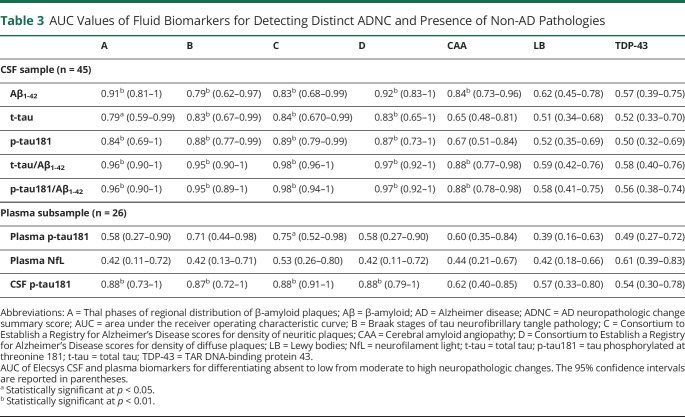
AUC Values of Fluid Biomarkers for Detecting Distinct ADNC and Presence of Non-AD Pathologies

Among common non-AD pathologies, the presence of CAA was highly associated with CSF Aβ_1-42_ levels (AUC 0.84 [0.73–0.96]) and with the t-tau/Aβ_1-42_ (AUC 0.88 [0.77–0.98]) and p-tau181/Aβ_1-42_ (AUC 0.88 [0.78–0.98]) ratios, but not with t-tau or p-tau181 levels individually (Table 3 and Figure 4). No CSF biomarker detected the presence of LB or TDP-43 pathology.

In the subsample with available plasma measurements, plasma p-tau181 was only significantly associated with Braak stage (B) (ρ = 0.43, *p* = 0.028) and neuritic plaque scores (C) (ρ = 0.47, *p* = 0.014), with corresponding AUC values of 0.71 [0.44–0.98] and 0.75 [0.52–0.98] (optimal cutoff 18.0 pg/mL), respectively (Figure 3 and Tables 2 and 3). Spearman correlations and AUC values of the associations between neuropathologic changes and Elecsys CSF biomarkers were comparable to the findings in the full CSF sample and were consistently higher for CSF p-tau181 than for plasma p-tau181. Plasma NfL levels did not show any association with ADNC scores or the presence of non-AD pathologies.

### Classification of Evidence

The primary objective of this study was to study the accuracy of antemortem Elecsys-derived CSF biomarkers to detect AD neuropathology as assessed by neuropathologic examination at autopsy. Our findings provide Class II evidence that the fully automated Elecsys-derived CSF t-tau and p-tau181 measurements, as well as their ratios with Aβ_1-42_ levels, discriminate between autopsy-confirmed AD and other dementias with high diagnostic accuracy (AUC 0.94–0.97).

## Discussion

In this study, we examined the association of Elecsys-derived CSF biomarkers for AD pathology and plasma measures of p-tau181 and NfL with neuropathologic changes at autopsy. Our findings demonstrate that Elecsys CSF biomarkers separated pathology-confirmed AD dementia cases from healthy controls and non-AD dementia cases with very high discriminative accuracy in vivo. In pathology-specific analyses, the individual Elecsys CSF Aβ and tau biomarkers showed strongest associations with the different AD neuropathologic measures that most closely reflect their pathologic target. Preliminary analysis of plasma p-tau181 in a smaller subset demonstrated comparable group separation accuracy and similar, albeit weaker, pathology-specific associations as CSF p-tau181. Taken together, our findings demonstrate the high neuropathologic validity and diagnostic accuracy of Elecsys CSF biomarkers and the potential of plasma p-tau181 as a cost-effective and scalable in vivo measure of AD pathology.

Fully automated CSF biomarker assays have recently been developed to satisfy the unmet need for laboratory- and batch-independent absolute CSF measures that will enable the use of universal biomarker cutoffs in both research and clinical settings.^5^ However, validation studies for the fully automated Elecsys CSF assays have so far covered only the concordance with Aβ-PET measures or clinical diagnostic and prognostic variables,^7-10,23^ leaving the neuropathologic validity of these automated biomarker measurements unclear. Here, we provide evidence of the very high diagnostic performance of Elecsys CSF biomarkers for discriminating between individuals with pathology-confirmed AD dementia and healthy controls and those with non-AD dementia. Notably, the diagnostic accuracy of the Elecsys-derived biomarkers was similar to or even higher than previously reported results for nonautomated CSF assays (see elsewhere^35^ for a recent meta-analysis), suggesting that this automatization did not result in lowered performance. Similar to previous findings on the diagnostic accuracy of individual CSF biomarkers, we found that CSF Aβ_1-42_ discriminated better between those with AD dementia and Aβ-PET–negative controls than CSF tau biomarkers,^19^ whereas CSF tau biomarkers discriminated better between AD and non-AD dementia cases than CSF Aβ_1-42_.^17,36^ The low CSF Aβ_1-42_ levels in the non-AD dementia group may partly be explained by comorbid Aβ pathology (3 of 4 cases in this group with CSF Aβ_1-42_ levels below the threshold also had intermediate or high A scores) but also may be affected by other pathologic or physiologic factors known to influence CSF Aβ_1-42_ levels.^27,37^ In both diagnostic contexts, the discriminative accuracy could be slightly improved with the use of the CSF tau-to-Aβ_1-42_ ratios.

Another key feature of our study was the evaluation of the relative sensitivity of Elecsys CSF biomarkers for different aspects of AD neuropathology. We found that although all Elecsys CSF biomarkers were strongly associated with the different ADNC scores, these associations were generally strongest between each biomarker and its respective target pathology (i.e., CSF Aβ_1-42_ vs Thal phase and diffuse plaques, and CSF p-tau181 vs Braak stage and neuritic plaques). These results are congruent with results from a previous study examining pathology-specific associations of CSF biomarkers measured by standard nonautomated assays in which pathologic measures of Aβ load were best correlated with CSF Aβ_1-42_ levels and pathologic measures of neurofibrillary tangle load were best correlated with CSF p-tau181 levels.^20^ However, in that study, the best neuropathologic correlate of both CSF biomarkers was CERAD neuritic plaque density. It also must be noted that CSF t-tau levels may be influenced by neurodegenerative processes such as neuronal death and axonal loss,^1-3^ which have not been assessed in the present study. Overall, the performance measures (correlation coefficients, AUC values) for the pathology-specific associations in that previous study were very similar to the ones observed here for the Elecsys CSF biomarkers, and the highest performance for pathology detection was also observed for the tau-to-Aβ_1-42_ ratios. Regarding non-ADNCs, we found that none of the Elecsys CSF biomarkers were associated with the presence of TDP-43 or LB pathology, but CSF Aβ_1-42_ levels were lower in cases with CAA. While this can be expected on the basis of the pathologic substrate of CAA and has been reported previously for a nonautomated CSF Aβ_1-42_ assay,^18^ the association between CSF Aβ_1-42_ levels and CAA pathology may also partly be explained by the high coprevalence of CAA and Aβ plaque pathology. Disentangling the Aβ pathology specificity of CSF Aβ_1-42_ levels would require larger and pathologically more heterogeneous study samples. Summarized, our results indicate high pathologic specificity of Elecsys CSF biomarkers for the different aspects of AD neuropathology and point to CAA as a potential confounder for the in vivo assessment of Aβ plaque pathology using CSF Aβ_1-42_ levels.

While CSF biomarker estimates from different assays are largely in agreement and highly correlated,^6,38^ they can show great differences in absolute quantifiable concentrations.^4^ This limits the development and application of universal abnormality thresholds for use across different laboratories and clinical settings. A key advantage of the Elecsys platform is its standardization through full automation of the assay, which has been proven to provide stable cutoffs for detecting PET-measured Aβ positivity and predicting clinical progression that generalize across cohorts.^7-9,23^ Nevertheless, no prior study has yet derived cutoffs for the Elecsys platform using a neuropathology gold standard. This is particularly relevant because derivation of CSF cutoffs based on clinical diagnosis has been shown to result in biased estimates due to misdiagnosis and the presence of concomitant pathologies.^36^ The pathology-based Elecsys cutoffs derived in this study are well within the range of previously established cutoffs based on correspondence to Aβ-PET or clinical endpoints. We found an optimal CSF Aβ_1-42_ cutoff of 1,097 pg/mL for discriminating high and low Thal phases, whereas high and low Braak stages and neuritic plaque scores were best separated by cutoffs of 229 and 19 pg/mL for t-tau and p-tau181, respectively (supplementary Table S1; data available from Dryad: doi.org/10.5061/dryad.n2z34tmwr). In comparison, optimal cutoffs for describing Aβ-PET positivity have been reported to range from 977 to 1,100 pg/mL for Aβ_1-42_, 213 to 242 pg/mL for t-tau, and 19 to 21 pg/mL for p-tau181.^7-9^ The strong agreement between our neuropathology analysis and these in vivo biomarker studies may be explained by the excellent accuracy of Aβ-PET for the detection of Aβ pathology,^24^ further supporting the generalizability of Elecsys CSF cutoffs across different research settings. ROC analyses for separating patients with pathology-confirmed AD dementia from Aβ-PET–negative healthy controls in our study yielded very similar t-tau (211 pg/mL) and p-tau181 (19 pg/mL) cutoffs compared to the pathology-specific cutoffs but indicated a considerably lower Aβ_1-42_ cutoff of 838 pg/mL (and thus higher cutoffs for the tau-to-Aβ_1-42_ ratios). This difference in the CSF Aβ_1-42_ cutoff can be expected because pathologic confirmation of AD requires the presence of both Aβ and tau pathologies and therefore implies more advanced disease stages that show lower average CSF Aβ_1-42_ levels.^9^

In preliminary findings from a smaller subsample analysis, we further compared the performance of Elecsys CSF biomarkers in the assessment of AD neuropathology with that of a novel plasma p-tau181 biomarker and with that of plasma NfL as a non–disease-specific neural injury marker. In line with recent neuropathologic studies of plasma p-tau181 and NfL biomarkers,^11,22,39^ both plasma p-tau181 and NfL demonstrated high accuracy for separating those with pathology-confirmed AD from healthy controls, but only p-tau181 demonstrated high accuracy in separating pathology-confirmed AD cases from non-AD dementia cases. The direct head-to-head comparison with CSF in our current study further indicated that the diagnostic accuracy of plasma p-tau181 was comparable to that of CSF p-tau181 in this differential diagnosis context. We also extended the existing neuropathologic studies on plasma p-tau181 measurements^11,22^ by investigating the specific neuropathologic correlates of this biomarker. Our analysis indicated a pathologic specificity of plasma p-tau181 for Braak tau stage and neuritic plaque scores similar to that of CSF p-tau181, although these associations were notably weaker, suggesting superior performance of Elecsys CSF biomarkers in this context.

Our study does have limitations. First, although relatively large for a combined antemortem CSF and postmortem neuropathology examination, the sample size of our study was still limited, particularly for the head-to-head comparison between CSF and plasma biomarkers. Moreover, the ADNI cohort represents a rather selective research cohort that may not reflect the general population, and the focus on autopsied individuals in this cohort introduces an additional selection bias, which is reflected in an older age, higher prevalence of men, and higher prevalence of an AD dementia diagnosis in our study sample (see supplementary Figure S1; data available from Dryad: doi.org/10.5061/dryad.n2z34tmwr). In addition, individuals with low levels of ADNC and non-AD dementia cases were underrepresented in our study; the sensitivity of the examined CSF and plasma biomarkers for early ADNC and their utility for differential dementia diagnosis thus remain to be established in more diverse cohorts. Cutoffs derived from small-sample analyses should be interpreted with caution. However, we are encouraged by the similarity between our cutoff values and those derived from larger studies using Aβ-PET or clinical outcomes as validation standards. Unfortunately, the CSF Aβ_1-42_/Aβ_1-40_ ratio, which has been proposed to compensate for individual differences in physiologic Aβ production,^37^ could not be assessed in our study. Moreover, the combination of plasma p-tau181 and Aβ markers may increase the correspondence with neuropathologic measures similar to the CSF p-tau181/Aβ_1-42_ ratio,^11,12^ but no measures of plasma Aβ were available for this study sample.

Our neuropathologic association study demonstrates high neuropathologic validity of Elecsys-derived CSF biomarkers of AD and further provides pathology-derived concentration cutoffs for this standardized analysis platform, which will support harmonization and interpretation of biomarker findings across different laboratories and clinical settings. In a smaller subset, our findings for plasma p-tau181 indicate similar, although weaker, pathology-specific associations with neuritic plaques and Braak tau stages as for CSF p-tau181. Its accuracy in discriminating between diagnostic groups adds strong support for the use of this easily accessible and scalable biomarker as a screening tool, particularly for the differential diagnosis of dementia. The performance of both Elecsys CSF and plasma p-tau181 measures as biomarkers of early-stage AD neuropathology remains to be investigated in larger and pathologically more diverse autopsy cohorts with available antemortem bodily fluid samples.

## Glossary

Aβ: β-amyloid
AD: Alzheimer disease
ADNC: AD neuropathologic change
ADNI: Alzheimer's Disease Neuroimaging Initiative
AUC: area under the curve
CAA: cerebral amyloid angiopathy
CERAD: Consortium to Establish a Registry for Alzheimer's Disease
IQR: interquartile range
LB: Lewy body
NfL: neurofilament light
NIA-AA: National Institute on Aging–Alzheimer’s Association
p-tau181: tau phosphorylated at threonine 181
ROC: receiver operating characteristic
t-tau: total tau
TDP-43: TAR DNA-binding protein 43
